# A human *MYBPC3* mutation appearing about 10 centuries ago results in a hypertrophic cardiomyopathy with delayed onset, moderate evolution but with a risk of sudden death

**DOI:** 10.1186/1471-2350-13-105

**Published:** 2012-11-10

**Authors:** Carolien H Teirlinck, Faïza Senni, Rajae El Malti, Danielle Majoor-Krakauer, Florence Fellmann, Gilles Millat, Xavier André-Fouët, François Pernot, Michaël Stumpf, Jean Boutarin, Patrice Bouvagnet

**Affiliations:** 1 Laboratoire cardiogénétique, Centre de Biologie et Pathologie Est, Groupe Hospitalier Est, 59 boulevard Pinel, Bron, Lyon, 69677, France; 2 Department Clinical Genetics of Erasmus Medical Centre Rotterdam, Rotterdam, the Netherlands; 3 Service de Génétique Médicale, CHUV, Lausanne, Switzerland; 4 Service de Cardiologie Pédiatrique, Hospices Civils de Lyon, Lyon, France; 5 Service de Cardiologie, Hospices Civils de Lyon, Lyon, France; 6 Service de Médecine Nucléaire, Centre Hospitalier de Valence, Valence, France; 7 Cabinet de Cardiologie, 31, rue Saint Maximin, Lyon, 69003, France; 8 Service de Cardiologie, Hôpital Saint Joseph, Saint Luc, Lyon, France; 9 EA 4173, Génomique fonctionnelle, Université de Lyon, Lyon, France

**Keywords:** Hypertrophic cardiomyopathy, MYBPC3, Mutation, Founder effect

## Abstract

**Background:**

Hypertrophic Cardiomyopathy (HCM) is a genetically heterogeneous disease. One specific mutation in the *MYBPC3* gene is highly prevalent in center east of France giving an opportunity to define the clinical profile of this specific mutation.

**Methods:**

HCM probands were screened for mutation in the *MYH7*, *MYBPC3*, *TNNT2* and *TNNI3* genes. Carriers of the *MYBPC3* IVS20-2A>G mutation were genotyped with 8 microsatellites flanking this gene. The age of this *MYBPC3* mutation was inferred with the software ESTIAGE. The age at first symptom, diagnosis, first complication, first severe complication and the rate of sudden death were compared between carriers of the IVS20-2 mutation (group A) and carriers of all other mutations (group B) using time to event curves and log rank test.

**Results:**

Out of 107 HCM probands, 45 had a single heterozygous mutation in one of the 4 tested sarcomeric genes including 9 patients with the *MYBPC3* IVS20-2A>G mutation. The IVS20-2 mutation in these 9 patients and their 25 mutation carrier relatives was embedded in a common haplotype defined after genotyping 4 polymorphic markers on each side of the *MYBPC3* gene. This result supports the hypothesis of a common ancestor. Furthermore, we evaluated that the mutation occurred about 47 generations ago, approximately at the 10th century.

We then compared the clinical profile of the IVS20-2 mutation carriers (group A) and the carriers of all other mutations (group B). Age at onset of symptoms was similar in the 34 group A cases and the 73 group B cases but group A cases were diagnosed on average 15 years later (log rank test p = 0.022). Age of first complication and first severe complication was delayed in group A vs group B cases but the prevalence of sudden death and age at death was similar in both groups.

**Conclusion:**

A founder mutation arising at about the 10th century in the *MYBPC3* gene accounts for 8.4% of all HCM in center east France and results in a cardiomyopathy starting late and evolving slowly but with an apparent risk of sudden death similar to other sarcomeric mutations.

## Background

Hypertrophic cardiomyopathy (HCM) is a frequent genetic disorder with an autosomal dominant inheritance. The prevalence of HCM is estimated at 1 in 500 in the general population [1]. It is likely that the prevalence of carriers with one of the genetic mutations that predispose to HCM is much higher, since the penetrance of the disease is variable and many asymptomatic carriers remain undiagnosed [2,3]. Sudden death can be the first symptom of the disease especially in young people where HCM is estimated an important underlying cause [4,5]. On the other hand, a proportion of carriers are asymptomatic and some HCM patients can have a normal life expectancy [6,7]. Sixty percent of HCM are caused by mutations in sarcomere genes. So far, defects in at least 13 different genes which encode a protein component of the sarcomere have been identified and more than 1000 different mutations within these genes were found [8-10]. Defects in the beta-myosin heavy chain (*MYH7*) and myosin-binding protein C (*MYBPC3*) genes are the most frequent causes for HCM. Defects in other genes like troponin T (*TNNT2*), troponin I (*TNNI3*), alpha-tropomyosin (TPM1), regulatory and essential myosin light chain (*MYL2*, *MYL3*), alpha-actin (*ACTC1*), titin (*TTN*) and alpha-myosin heavy chain (*MYH6*) are less frequent causes for HCM [8-10].

A great variation in age at onset and severity of features of HCM has been observed between HCM patients with sarcomere gene defects. Elucidation of phenotype/genotype correlation of HCM is of special interest because this may allow risk stratification according to genetic defects. The study of genotype/phenotype correlation has been hampered because of the many different mutations and low frequency of each mutation. It is also believed that there are genetic modifier factors that either compensate or aggravate the impact of the causal mutation [11]. In center east of France, 9 families with more than 30 carriers of the same genetic mutation (GenBank: NM_000256.3 c.1928-2A>G or IVS20-2A>G) in the *MYBPC3* gene are currently followed at the Groupe Hospitalier Est in Lyon. In this report, we demonstrated that this was a founder mutation and we further detailed the clinical profile resulting from this mutation with delayed age at diagnosis despite early symptoms, a slow progression toward complications but a risk of sudden death similar to carriers of other sarcomeric mutations.

## Methods

### Ethics statement

Before proceeding to blood drawing, informed consent was signed by the patient or by parents. The informed consent was requested for diagnosis and for research purposes. Only blood samples which were obtained with an agreement for both diagnosis and research were used in this study. This procedure complies with the current laws in France (Loi Biomédecine 2004) and the last version of the Declaration of Helsinki (The World Medical Association, 2008).

### Study population

The diagnosis of Hypertrophic Cardiomyopathy (HCM) was established according to international criteria [12]. Patients diagnosed with hypertrophic cardiomyopathy, whatever their age, were proposed to have a visit with a geneticist in order to obtain family medical history and to propose a genetic screening of the 4 following sarcomeric genes: *MYBPC3*, *MYH7*, *TNNT2* and *TNNI3*. The pathogenicity of variants was inferred from several criteria: conservation of the amino-acid, location in a protein domain, absence from controls (200 healthy ethnic matched controls, 1 000 genomes and the Exome Variant Server), published mutation. If a mutation was found, it was systematically confirmed by a second sample and relatives were systematically proposed to be tested for their status of carrier or non-carrier. In any case, patients received genetic counseling.

**Figure 1:**
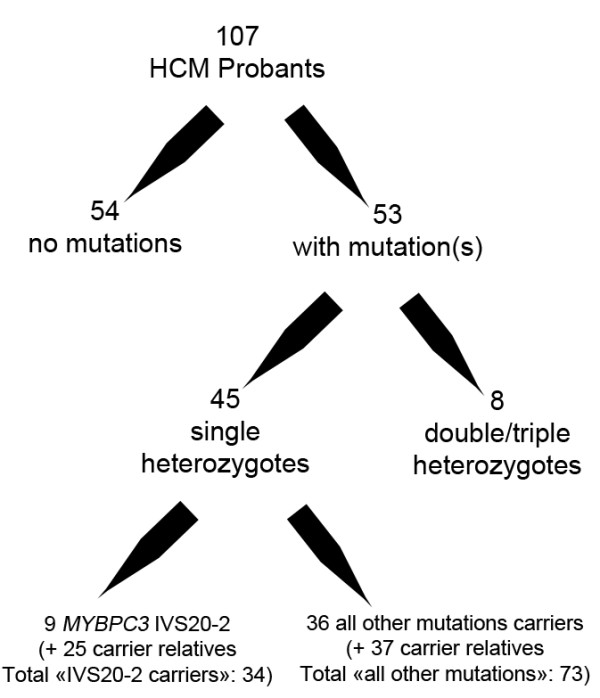
Scheme summarizing the sorting process of HCM cases.

We selected all persons with a mutation in one of the following genes *MYBPC3*, *MYH7*, *TNNT2* or *TNNI3* (HCM patients and mutation carrier relatives whether they were symptomatic or not). Among 107 HCM patients, a mutation was found in 53 cases. Eight HCM cases were excluded because they were double (7 cases) or triple (1 case) heterozygotes. Among the 45 single heterozygote HCM cases, 9 had the same *MYBPC3* mutation (IVS20-2A>G). A mutation screening was proposed to relatives irrelevant to age, clinical status and mutation. Mutation carriers were classified into one group of 34 carriers of the *MYBPC3* IVS20-2A>G mutation (group A) and 73 carriers of any other single mutation in the *MYBPC3*, *MYH7*, *TNNT2* or *TNNI3* genes (group B). This latter group included 15 *MYBPC3* probands (other than the IVS20-2A>G mutation), 16 *MYH7*, 2 TNNT2 and 3 *TNNI3* probands (Figure 1).

### Genotyping

The *MYBPC3* gene is located on the short arm of chromosome 11 close to the centromere from nt 47 352 957 to nt 47 374 253 according to Ensembl database. Four polymorphic markers on each side of the *MYBPC3* gene were selected: D11S1763, D11S986, D11S4137 and D11S1344 on the telomeric side and D11S1784, D11S1326, D11S4165 and D11S1765 on the centromeric side. These polymorphic markers are at genomic position 42 861 500 nucleotides (nt), 44 722 300 nt, 45 601 500 nt and 46 167 00 nt on the telomeric side and 48 022 900 nt, 49 324 500 nt, 50 138 000 nt and 66 778 544 nt on the centromeric side of the *MYBPC3* gene, respectively. All IVS20-2 *MYBPC3* mutation carriers were genotyped. In addition, the DNAs of 50 controls of the same European origin were genotyped at these 8 polymorphic markers to evaluate the distribution of alleles in the general European population. Genotyping was performed with the universal fluorescent labeling method [13]. In brief, a tag sequence (5^′^ – GGTGGCGACTCCTGGAG – 3^′^) was added to the 5^′^-end of the forward primer. A fluorescent-labeled primer (FAM – GGTGGCGACTCCTGGAG – 3^′^) was added to the PCR reaction. PCR reaction were obtained using 10 ng of genomic DNA, 10 mM Tris–HCl (pH 8.8), 50 mM KCl, 1.5 mM MgCl2, 100 μM deoxynucleotide triphosphate, 5 pmol of each tagged forward primer and FAM-labeled reporter primer, 10 pmol of reverse primer and 0.5 units of Taq polymerase in a total volume of 20 μl. Cycling conditions were 94°C for 60 sec, 60°C for 60 sec, and 72°C for 60 sec in a Robotcycler (Stratagene, Netherland). PCR products were electrophoresed in an ABI 3100 system (Applied Biosystems, Foster city, CA), and the data were analyzed using GeneScan Analysis Software version 2.1 (Applied Biosystems).

### Haplotype reconstruction and search for a common ancestor

Haplotypes were reconstructed starting with the family with the largest number of mutation carriers. Allele frequency was estimated on a group of 50 controls. The Genethon and the deCode genetic maps were used to infer the position of the 8 polymorphic markers. Recombination fractions between the different markers and the *MYBPC3* gene were then computed from genetic distances using Kosambi mapping function assuming that the correlation between genetic and physical distances between the two closest markers to *MYBPC3* was constant in this interval. We then used the ESTIAGE program [14] to estimate the age of the most recent ancestor of this mutation. A mutation rate of 10^-4^ per individual per generation and a stepwise mutation model was assumed at the polymorphic markers. Patients were assumed to carry the ancestral haplotype on each side of the mutation up to the first marker where they do not show an allele similar to the ancestral allele at this locus.

### Design and setting of clinical profiling

We performed a retrospective cohort study. Information was retrieved from the medical records. Family histories were ascertained during genetic consultations. In addition for this study, all participants were contacted by telephone and administered a semi-structured questionnaire. With consent of the participants, their cardiologists were requested to fill in a semi-structured questionnaire asking for an update about follow-up and the results of clinical tests like echocardiograms and electrocardiograms. Participants and their cardiologists were asked to send copies of reports on medical examinations, electrocardiograms (at rest and ambulatory), and echocardiograms. About half of the participants are jointly followed by their local cardiologist and our center. Thus, we could also get directly information from our center. Family histories were evaluated for the occurrence of sudden death.

### Statistical data analysis

We used SPSS version 15.0 to analyze the data. Comparisons were made with Student’s *t*-test and Chi Square. For time free from clinical event analysis, we created Kaplan-Meier curves and comparisons of time free from clinical event curves between groups were performed with a log rank test. To evaluate the prevalence of sudden death, we recorded all sudden death in each family among all family members reported during family history registering.

## Results

### Study population

Carriers of the *MYBPC3* IVS20-2A>G mutation were assigned as group A and the carriers of one of the other mutations in the examined sarcomere genes *MYBPC3*, *MYH7*, *TNNT2* and *TNNI3*, were group B cases. Altogether, 107 subjects from 45 different families were included. There were 34 *MYBPC3* IVS20-2A>G carriers (group A) from 9 apparently unrelated families and 73 all other mutations carriers from 36 families (group B) (Figure 1). The mutations of group B cases are presented in Table 1. All but 2 of these 29 different mutations were already reported by Millat et al. (2010) [15]. One of these 2 mutations is the mutation MYH7 p.Arg453His which has already been reported by Yu et al. [16] and the other is the in-frame deletion of 4 highly conserved amino-acids (p.1101_1104delGSQL) at the beginning of the myosin tail. Three mutations in *MYH7* (p.Thr177Ile, p.Asn696Ser and p.Ile1927Phe) were previously reported by Millat et al. (2010) [15] in HCM patients carrying also other mutations. In the current report, only individuals carrying a single heterozygous mutation were included. All 3 amino-acids are highly conserved and predicted as disease causing and deleterious (Mutation Taster and SIFT). In addition, the *MYH7* p.Asn696Ser mutation was already reported by Jaaskelainen et al. [17]. None of these 5 mutations were found in the SNP databases and in 200 ethnically matched controls.

**Table 1:** Mutation description of group B cases (All other mutations)

**Gene**	**Mutation**	**Number of carriers**
MYBPC3	p.Tyr79X	5 (1)
MYBPC3	p.Arg272Cys	1 (1)
MYBPC3	IVS7+5G>A	6 (3)
MYBPC3	p.Phe305fs	1 (1)
MYBPC3	IVS12-2A>G	1 (1)
MYBPC3	IVS13+1G>A	1 (1)
MYBPC3	p.Arg495Gly	2 (1)
MYBPC3	p.Ala701Thr	1 (1)
MYBPC3	p.Arg943X	2 (2)
MYBPC3	p.Gln969X	2 (1)
MYBPC3	p.Ile1131Thr	1 (1)
MYBPC3	p.Cys1244X	5 (1)
MYBPC3	p.Tyr1251X	1 (1)
**MYH7**	**p.Thr177Ile**	**5 (1)**
MYH7	p.Arg403Gln	1 (1)
MYH7	p.Val411Ile	3 (1)
**MYH7**	**p.Arg453His**	**1 (1)**
MYH7	p.Val606Met	6 (2)
**MYH7**	**p.Asn696Ser**	**1 (1)**
MYH7	p.Arg719Trp	1 (1)
MYH7	p.Arg719Gln	3 (1)
MYH7	p.Val878Gly	3 (1)
**MYH7**	**p.1101_1104delGSQL**	**3 (1)**
MYH7	p.Arg1420Trp	4 (2)
**MYH7**	**p.Ile1927Phe**	**3 (1)**
TNNT2	p.Ala157Ser	1 (1)
TNNT2	p.Arg278Cys	2 (2)
TNNI3	p.Arg136Gln	1 (1)
TNNI3	p.Lys183Asn	6 (2)

Bold letters for mutations not reported by Millat et al. 2010 [15] or mutations reported in double heterozygotes ou compound heterozygotes by Millat et al. 2010 [15]. Numbers in parenthesis: number of families.

There was no difference in gender between group A and B mutation carriers (p = 0.13). The same percentage of group A and group B cases were diagnosed with HCM (62%, n = 21) and (67%, n = 49) (p = 0.59), respectively (Table 2). In total 23 (68%) group A cases and 49 (67%) group B cases reported symptoms (p = 0.96), including dyspnea, fatigue, palpitations, syncope (malaise and/or loss of consciousness) and thoracic pain (specific and non-specific) or were said to have a heart murmur. The average age at last visit was 47 years in group A and 39 years in group B (p = 0.045).

**Table 2:** Demographic data on the groups A (MYBPC3 IVS20-2A>G) and B (all other mutations carriers)

	**Group A**	**Group B**
Subjects (n)	34	73
Families (n)	9	36
Women (n)	23 (67.6%)	38 (52.1%)
Patients with HCM (n)	21 (61.8%)	49 (67.1%)
Symptomatic carriers (n)	23 (67.6%)	49 (67.1%)
Average age at last visit (year)	47.28*	39.07

*: p < 0.05.

### Founder effect

All IVS20-2 mutation carriers were genotyped for markers D11S1763, D11S986, D11S4137 and D11S1344 on the telomeric side of *MYBPC3* and D11S1784, D11S1326, D11S4165 and D11S1765 on the centromeric side. Because *MYBPC3* is located close to the centromere, we selected the most distal polymorphic marker (D11S1765) on the other side of the centromere (hence in the long arm of chromosome 11) at a physical distance of more than 13 million nucleotides. Physical and genetic map positions are provided on Table 3. Physical positions are referring to chromosomal positions whereas genetic positions are given relative to the *MYBPC3* gene. The complete haplotype of the mutation could be inferred in 7 of the 9 families (F01-F05, F07 and F08) and in 2 families the allele associated with the IVS20-2 could not be determined at a single polymorphic marker (families F06 and F09) (Table 4) because mutation carriers had the same 2 alleles. On the centromeric side, all IVS20-2 mutation carriers had the same allele at the 3 closest markers to *MYBPC3*: 160 (D11S1784), 268 (D11S1326), 237 (D11S4165) (from the *MYBPC3* gene toward the centromere) but 5 of the 9 possible alleles were found on the most distal marker (D11S1765). On the telomeric side, all families had the same allele only at the closest polymorphic marker to *MYBPC3*: 293 (D11S1344). The next tested polymorphic marker away from the *MYBPC3* gene was D11S4137. Families F01 to F05 had allele 298, while families F07 to F09 had the allele 284. Family F06 was too small to determine the allele associated with the IVS20-2 mutation. The allele could be 284 or 298 (Table 4). For the most distant markers on the telomeric side (D11S986 and D11S1763), 6 out of 13 possible alleles and 4 out of 5 possible alleles were found, respectively. These data demonstrate that the haplotype carrying the IVS20-2 *MYBPC3* mutation was the same in all 9 families lending support to the hypothesis of a founder mutation. Furthermore, it suggested a phylogenic tree between IVS20-2 families with 2 main branches one carrying the 284 allele and the other the 298 allele at marker D11S4137. Families F03 and F04 shared the same haplotype on 7 of the 8 analyzed polymorphic markers, while families F05 and F06 might also share a common haplotype on 7 of the 8 markers. The approximate time of occurrence of this mutation was inferred by the ESTIAGE software taking into account the genetic distance between polymorphic markers and the prevalence of alleles associated to IVS20-2 mutations. The estimation was that the mutation occurred 47 generations (CI 95%, 27 – 65) ago. With an average generation every 20–25 years, the mutation appeared approximately 10 centuries ago (time range from the 4^th^ to the 15^th^ century). Since IVS20-2 mutations carriers of these 9 families were actually descendants of a common ancestor, they all carried the same haplotype on the chromosomal region adjacent to the IVS20-2 MYBPC3 mutation. A genetically homogenous population of HCM patients provides an opportunity to evaluate the clinical profile of the cardiomyopathy caused by this mutation.

**Table 3:** Physical and genetic positions of the polymorphic markers flanking the MYBPC3 gene used in this study

**AFM name**	**D name**	**Physical position (nucleotides)**	**Genetic position (cM)**
AFM162xg1	D11S1763	42 861 500	4.92
AFM255ye1	D11S986	44 722 300	2.06
AFMb036ya9	D11S4137	45 601 500	0.48
AFM298vc9	D11S1344	46 167 000	0.14
Mutation		47 361 363	0.00
AFMa139yb9	D11S1784	48 022 900	0.16
AFM255zg1	D11S1326	49 324 500	0.17
AFMb333ye1	D11S4165	50 138 000	0.23
AFM165zc3	D11S1765	60 778 500	2.28

**Table 4:** Haplotypes associated with the IVS20-2 mutation in the 9 families (F01 to F09)

	**F01**	**F02**	**F03**	**F04**	**F05**	**F06**	**F07**	**F08**	**F09**
**D11S1763**	201	199	201	201	201	201	203	199	197
**D11S986**	180	178	176	176	174	174	152	152	170/174
**D11S4137**	298	298	298	298	298	298/284	284	284	284
**D11S1344**	293	293	293	293	293	293	293	293	293
**MYBPC3**	M	M	M	M	M	M	M	M	M
**D11S1784**	160	160	160	160	160	160	160	160	160
**D11S1326**	268	268	268	268	268	268	268	268	268
**D11S4165**	237	237	237	237	237	237	237	237	237
**D11S1765**	262	262	260	249	249	259	259	259	253

*M*: mutation.

### Diagnosis time and symptoms

**Figure 2:**
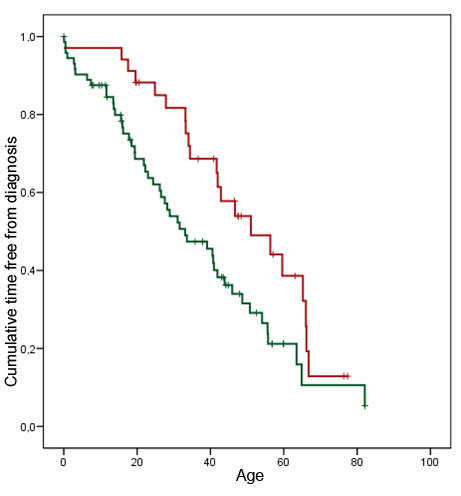
**Age at diagnosis of hypertrophic cardiomyopathy.** Time free from diagnosis curve. Red graph: group A cases (*MYBPC3* IVS20-2A>G mutation carriers); green graph: group B cases (all other mutation carriers). Log rank test p = 0.022.

**Figure 3:**
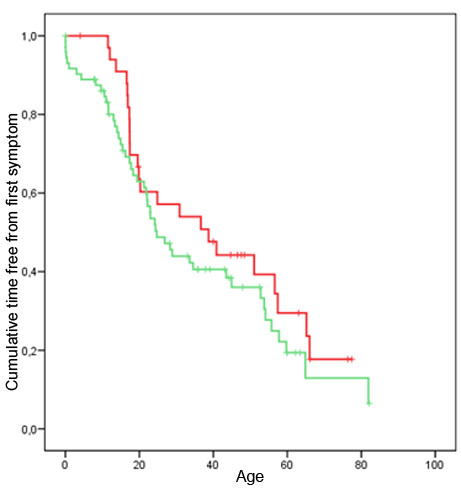
**Age at first symptom.** Time free from first symptom curve. Red graph: group A cases (*MYBPC3* IVS20-2A>G mutation carriers); green graph: group B cases (all other mutation carriers). Log rank test p = 0.226.

The thickening of the ventricular septum to 13 mm was reached on average 15 years later in life in group A than in group B (Figure 2) log rank test p = 0.022. This figure is interesting for 2 points that need to be detailed. First, it clearly shows that the delay in the appearance of hypertrophy between groups A and B was constant during life span since the 2 curves are parallel. Second, it is also obvious for group A cases as well as for group B that the disease penetrance increased at a regular pace during life span since the 2 curves are nearly straight. By contrast, the age at time of first symptoms was similar in cases of group A and B (Log rank test p = 0.226), (Figure 3). The frequency and nature of first symptoms as fatigue, palpitations or thoracic pain was similar between group A and B although dyspnea was reported slightly more frequently as a first symptom in group B. Among group A cases, there was a slight excess of patients reporting to be diagnosed previously with a murmur or to have suffered syncope. Interestingly, in group B, first symptom and diagnosis of HCM occurred almost at the same time (Figure 3) whereas diagnosis occurred several years after onset of first symptoms in group A (Figure 2).

### Complications

**Figure 4:**
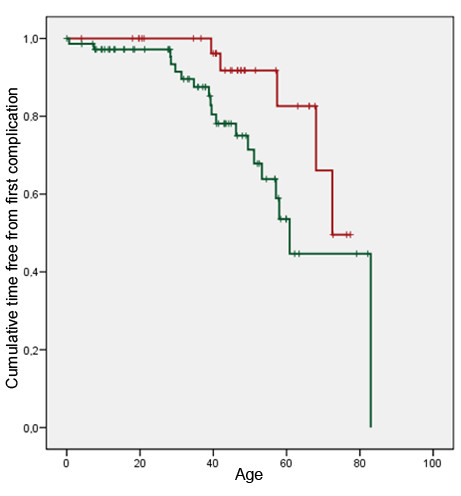
**Age at first complication or therapeutic outcome.** Time free from first complication curve. First complication or therapeutic outcome: any of the following events: external cardioversion, pacemaker implantation, interventricular wall thinning (alcohol or surgery), defibrillator implantation, heart failure, heart transplantation, cardiac death. Red graph = group A cases; green graph = group B cases. Log rank test p = 0.047.

**Figure 5:**
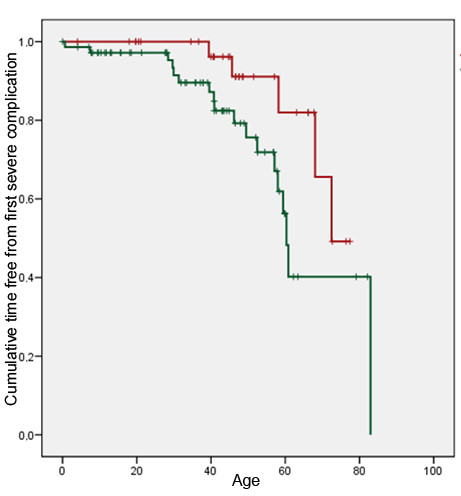
**Age at first severe complication or severe therapeutic outcome.** Time free from first severe complication curve. Severe complication or severe therapeutic outcome: any of the following events: interventricular wall thinning (alcohol or surgery), defibrillator implantation, heart failure, heart transplantation, cardiac death. Red graph = group A; green graph = group B. Log rank test = 0.059.

The following complications and therapeutic outcome related to hypertrophic cardiomyopathy were registered: external cardioversion, pacemaker implantation (both events considered as moderate events), heart failure, interventricular wall thinning (surgical or alcohol), defibrillator implantation, heart transplantation or cardiac death (complications or therapeutic outcome considered as severe). No subjects died between the time of molecular diagnosis and the end of the study. Five group A cases (15%) and 19 group B cases (26%) had complications. Although the difference in overall complications and therapeutic outcome was not significant (p = 0.19) between groups A and B, there is an excess of implantation of pacemakers and defibrillators, heart transplantations and heart failure among the group B cases (Table 5). Furthermore, group A cases were approximately 15 years older than group B cases when first complications of HCM occurred (any moderate or severe event) (Figure 4) (log rank test p = 0.047). When comparing groups A and B about the age of occurrence of severe complications or therapeutic outcome (heart failure, interventricular wall thinning, defibrillator implantation, heart transplantation, and cardiac death) the curves were still separated but no longer statistically different (log rank test p = 0.059). The approximate difference in age of severe complications or severe therapeutic outcome between groups A and B was only about 10 years (Figure 5). We examined the family history of the participants to investigate if the prevalence of sudden death (SD) was higher in group A families than group B families. In group A, SD was reported 14 times in 9 families with 292 relatives whereas in group B, SD was reported 25 times in 36 families with 837 relatives. When we compared the prevalence of SD in group A families (14/292 = 0.048) and group B families (25/837 = 0.030) the prevalence of SD was slightly higher in group A then in group B but the relative risk was not statistically different: RR 1.6 95% CI 0.85-3.05. The mean age at SD was statistically not different in group A vs group B, 46.6 y. and 43.1 y., p = 0.59, respectively.

**Table 5:** complications in group A and B cases

	**Cardioversion**	**Pacemaker**	**Defibrillator**	**Wall thinning**	**Heart failure**	**Heart transplantation**
Group A (n)	2 (5.9%)	1 (2.9%)	1 (2.9%)	2 (5.9%)	2 (5.9%)	0
Group B (n)	5 (6.8%)	4 (5.5%)	8 (11.0%)	4 (5.5%)	11 (15.1%)	5 (6.8%)

## Discussion

This study clearly demonstrates that the *MYBPC3* IVS20-2 mutation is a founder mutation in our cohort and not a hot spot of mutation. Hot spots of mutation are regions of DNA that are “fragile” and often mutated. In this latter case, mutations appear independently at different time and in different individuals. Although, these individuals share the same mutation, they do not share the same alleles in the adjacent DNA region. By contrast, descendants of a mutation carrier will share not only the mutation but also the alleles in the adjacent genomic region. This is true for the closest DNA variants to the mutation but less true for distant variants because the probability that a mutation carrier has also the founder allele at remote positions decreases with genomic distance. This is due to the fact that the probability of the occurrence of a recombination increases with the genomic distance between the mutation and the variant. A founder mutation was already evidenced in the *MYBPC3* gene in other populations [18-21]. These founder mutations account for 24% (Netherland [18]) to less than 15% (Germany [19], Finland [22], Japan [20], Spain [21]) of HCM mutations. The IVS20-2 *MYBPC3* mutation in our cohort accounts for 8.4% of unselected HCM and 18% of HCM with at least a mutation in the 4 tested sarcomeric genes. These founder mutations are located in different gene regions but all predict a C-terminal truncated protein due to a premature termination codon: a nonsense as in Finland (Gln1061X) and Turkey [19] or a frameshift as in Netherland (2373insG) and Japan (c.11645delT, p.V592fs8X); or an altered splicing as in Spain (IVS23+1G>A), in Germany [19] and in France (IVS20-2A>G). In that respect, they are not different from the other MYBPC3 mutations that lead in 80% of the cases to premature termination of translation either by introducing a stop codon, a frame-shift or an altered splicing.

Since our patients not only carry the same *MYBPC3* mutation but also the same variants within the *MYBPC3* gene sequence and flanking DNA, the expression profile of the mutation depends only on the other MYBPC3 gene copy and on distant genetic factors giving an opportunity to observe a specific clinical profile. Disease signs in IVS20-2 *MYBPC3* carriers appear at the same age as in carriers of other sarcomeric mutations but the diagnosis was delayed by approximately 15 years suggesting that the electric and echocardiographic hypertrophy is delayed in IVS20-2 carriers (group A). Interestingly, the curves on first symptom and diagnosis age show that there is no age threshold but rather a regular increase in penetrance of the disease. A 15-year delay in the evolution towards the first complications is also demonstrated when IVS20-2 *MYBCP3* mutation carriers are compared to other mutation carriers. This relatively benign evolution fades when focusing on severe complications and disappears when concentrating on the risk of sudden death. By contrast, Millat et al. ([2010]) [15] reported a higher sudden death ratio in HCM probands with an identified mutation compared to HCM probands with no identified mutation. No systematic autopsy was performed in these sudden death cases. Consequently, we cannot be certain that every sudden death case is secondary to hypertrophic cardiomyopathy but there is no reason to believe that sudden death cases of other origin are more prevalent in one group than in the other. A previous study in the Netherlands showed that *MYBPC3* truncating mutations had mild effects in the first three decades of life, while the phenotype later in life is severe [23]. The relative high risk of sudden death contrasting with a benign and delayed natural history was also observed in the Japanese study [20] with 13% of sudden death over a mean follow-up of 9 years and in the Spanish study [21] with 17 sudden deaths in 12 families. It might seem surprising that mutations resulting in a high risk of sudden death can nevertheless become a founder mutation. Dominant mutations are generally responsible for a negative pressure of selection and tend to disappear after several generations because of this survival decrease of mutation carriers. Founder mutations escape this pressure of selection and are transmitted along generations. The reason for this exception is well demonstrated in this report since the expression of the disease is delayed beyond the reproductive age. Hence, when the disease is responsible for complications, the patient has already transmitted the mutation to his/her descendants. When comparing the clinical profile of HCM depending of the involved genes, one should keep in mind that the percentage of founder mutation carriers might soften the profile. For instances, in Finland or in The Netherlands where about 24% of *MYBPC3* mutation carriers have a founder mutation, the mean MYBPC3 clinical profile can only be mild. Does this hold true when founder mutation carriers are removed? In particular, it would be interesting to study separately *MYBPC3* missense mutations that could result in HCM clinical profile comparable to mutations in the *MYH7* gene.

## Conclusion

The IVS20-2A>G mutation in the 9 families followed at the department of cardiogenetics in Lyon, is a founder mutation since mutation carriers also share the adjacent genomic region. Carriers of the IVS20-2A>G mutation in the *MYBPC3* gene were diagnosed later in life than carriers with other mutation in the *MYBPC3*, *MHY7*, *TNNT2* and *TNNI3* genes, despite the fact that they experienced first symptoms at the same age. The delay in diagnosis in this particular group of patients is probably due to a delayed appearance of hypertrophy, leading to later diagnosis. Accordingly, complications were observed later in life in HCM patients with the IVS20-2 mutation than in HCM patients with other mutations. Nevertheless, the IVS mutation was associated with the same high rate of SD as carriers of other mutations. Therefore, despite the late onset and the delayed evolution towards complications, our study shows that cardiologists should keep in mind a risk of SD in families with the *MYBPC3* IVS20-2A>G mutation.

## Competing interests

The authors declare that they have no competing interests.

## Authors’ contributions

CTH was in charge of collecting data, FS and REM carried out the haplotype study, CTH, DMK and PB performed the statistical analysis, FF, XAF, FP, MS and JB contributed HCM cases and participated in the design of the study, GM performed mutation screening, PB conceived the study, all authors contributed to write the article. All authors read and approve the manuscript.

## Pre-publication history

The pre-publication history for this paper can be accessed here:

http://www.biomedcentral.com/1471-2350/13/105/prepub
